# Editorial: Nutrient Dependent Signaling Pathways Controlling the Symbiotic Nitrogen Fixation Process

**DOI:** 10.3389/fpls.2021.744450

**Published:** 2021-09-08

**Authors:** Takuya Suzaki, Vladimir Totev Valkov, Maurizio Chiurazzi

**Affiliations:** ^1^Faculty of Life and Environmental Sciences, University of Tsukuba, Tsukuba, Japan; ^2^Institute of Biosciences and Bioresources (IBBR), Italian National Research Council (CNR), Napoli, Italy

**Keywords:** transporters, transcription factors, nitrate, nitric oxide, miR2111, phosphate

Plants evolved an amazing capacity to cope with sudden changes in environmental conditions. In the case of changes in nutrient availability, the optimal response is achieved through a cross-talk between the perception of an external signal with the endogenous nutritional status and plant demands. Symbiotic Nitrogen Fixation (SNF) is a paradigmatic example of this signaling network controlling formation, development, and functioning of N_2_-fixing nodules.

Nitrate is a well-studied signal regulating the early step of nodule formation through local and systemic pathways (Omrane and Chiurazzi, 2009). Legumes possess a systemic regulatory system called autoregulation of nodulation (AON), which not only plays a central role in maintaining an optimal number of nodules but it is also involved in controlling nodulation in response to nitrate (Nishida and Suzaki, 2018). The microRNA 2111 (miR2111) was recently identified as a key mobile factor of AON translocated from shoot (leaves) to roots where it positively regulates nodulation by inhibiting the role of its target TOO MUCH LOVE (TML), an F-box protein with a negative role in nodulation (Tsikou et al., 2018; Okuma et al., 2020). The initial step of AON is induced in legume roots by rhizobial infection or high soil nitrate concentrations, through the induction of the CLAVATA3/ENDOSPERM SURROUNDING REGION (CLE)-related mobile signals (Reid et al., 2011). CLE peptides are traslocated to the shoot where they activate a leucine-rich repeat receptor-like kinase (NODULE AUTOREGULATOR RECEPTOR KINASE, NARK in soybean; Reid et al., 2011). NARK then negatively regulates miR2111 production which leads to enhanced production of TML and inhibition of nodulation, resulting in an optimal number of nodules. In this Research Topic, Okuma and Kawaguchi overview the latest findings on the function of miR2111 and its upstream and downstream molecular mechanisms of AON. Furthermore, they expand the scope of discussion beyond root nodule symbiosis, by discussing the potential roles of miR2111 in plant-microbe symbioses other than root nodule symbiosis and in nutrient signaling of non-nodulating plants. As they mention, miR2111 was originally identified as a phosphate (Pi)-responsive miRNA in Arabidopsis, and the implications of Pi-deficiency in AON have been recently shown (Isidra-Arellano et al., 2020). It will be an intriguing research subject to reveal the conserved and diversified mechanisms that underlie systemic control of organogenesis and nutrient signaling by focusing on the role of miR2111 in nodulating and non-nodulating plants.

The AON and the nitrate-dependent pathway of inhibition of nodulation are also reviewed by Ma and Chen. The transcription factor Nodule INception (NIN) is a well-known central regulator of the root infection and nodule organogenesis programs. The authors focus the attention on the role played by the NIN-like proteins (NLP) in the ${\text{NO}}_{3}^{-}$ dependent inhibition of nodule development by describing their structural specificity and mode of action. Furthermore, Ma and Chen report in their review different aspects of the nitrate- and ammonium-dependent inhibitory pathways of nodule formation. They describe the nodular expression patterns of a large number of the nitrate transporter peptide family (NPF) and high affinity nitrate transporter (NRT2) members, as well as the ammonium transporters belonging to the AMT1 and AMT2 families and their involvement in the nodulation process. The authors also discuss the phosphate signaling pathways controlling nodulation efficiency. A sufficient supply of Pi is required for efficient nodule development and symbiotic N_2_-fixation. The network of transporters and transcription factors involved in this Pi-dependent signaling pathway is reported. Interestingly, Pi and ${\text{NO}}_{3}^{-}$ signaling pathways appear to be based on partially overlapping mechanisms (Isidra-Arellano et al., 2020). In particular, the authors focus on the similarities between the pathways governing hormonal distribution in roots for the control of nodule formation and on the mechanisms of N-feedback regulation (through asparagine accumulation) controlling the autoregulatory systemic pathway and the nitrogenase activity. The crosstalk between ${\text{NO}}_{3}^{-}$ and Pi signaling is also suggested by the analyses of the transcriptional regulation triggered by phosphate starvation and nitrate response.

Villar et al., report an in-depth molecular and biochemical characterization of a peculiar *Medicago truncatula* non-symbiotic hemoglobin of class 1, encoded by the *MtGlb1-2* gene. Unlike any other hemoglobin gene characterized so far (Becana et al., 2020), the precursor RNA of *MtGlb1-2* gives rise to four alternative splice forms, which are named here *MtGlb1-2.1* to *MtGlb1-2.4*. The different splice forms, including those encoding for long proteins with two heme groups, are predominantly expressed in the meristems and vascular bundles of roots and nodules. Furthermore, *MtGlb1-2* is transcriptionally induced by hypoxia and NO sources such as ${\text{NO}}_{3}^{-}$ or *S*-nitrosoglutathione (GSNO). An interdisciplinary approach, exploiting biochemical and biophysical methods, allowed them to investigate the reactivity toward O_2_, NO, and ${\text{NO}}_{2}^{-}$ of the two MtGlb1-2.1 and MtGlb1-2.4 *in vitro* produced proteins, representing the isoforms with two or one heme, respectively. The extremely high reactivity of the two isoforms toward physiological ligands of Glbs such as O_2_, NO, and nitrite was demonstrated through analyses of O_2_ affinity, NO binding capacity as well as nitric oxide dioxygenase (NOD) and nitrite reductase (NiR) activities. The latter two enzymatic activities reported for MtGlb1-2 occur at opposite O_2_ levels, as the NOD activity requires O_2_ and scavenges NO, whereas the NiR activity requires anaerobic (or nearly) conditions and generates NO. Villar et al., propose that MtGlb1-2 acts in the meristems and vascular bundles of roots and nodules either as a NO scavenger or NO producer, depending on the O_2_ tension in the plant tissue, for a fast and fine tuning of NO concentration in the cytosol in response to rapid changes in O_2_ availability.

The data provided by Villar et al., track an interesting link with some recent reports highlighting the role of nitrate (the main source of NO in plants) and nitrate transporters in the control of nodule function efficiency. In particular, members of the NPF and NRT2 families have been reported to play a positive role on nodule functioning by controlling the quick and fine tuning of nitric oxide (NO) concentration in the nodule-invaded cells in response to rapid changes in O_2_ availability (Valkov et al., 2020; Wang et al., 2020). In this Research Topic, Vittozzi et al. report the characterization of another *L. japonicus* NPF member, *LjNPF3.1*, whose expression is induced in mature nodules, which could be involved in that nitrate-dependent pathway. In particular, the pattern visualized in hairy roots transformed with a *proNPF3.1:tYFP-NLS* reporter construct localizing triple YFP in the nucleus of transgenic roots, indicates an expression localized in the periphery of the nodule organ. As represented in the scheme illustrated in Figure 7 of Vittozzi et al., this spatial pattern of expression could be involved in the flow of nitrate toward nodule vascular bundles and N_2_-fixation zone where the fine tuning of nitric oxide concentrations can be required in response to changes of O_2_ pressure. The positive role played by *LjNPF3.1* is indicated by the reduced N_2_-fixation activity associated to stress-related anthocyanin accumulation, displayed by two independent null mutants isolated from the LORE1 collection (Malolepszy et al., 2016). However, in the case of LjNPF3.1, a direct evaluation of the transported substrate has not been provided and an intriguing role as a gibberellin (GA) transporter based on the reported features of other members of the clade 3 of the NPF family cannot be excluded.

In this Research Topic, another research team contribution to the analysis of the role played by NPF genes in the regulation of nodulation and symbiotic nitrogen fixation is provided in the article by Yu et al. MtNPF1.7 plays a role in nodule development as well as lateral and primary root growth (Yendrek et al., 2010). Despite the potential significant role of MtNPF1.7 for the regulation of such developmental programs, its functional and structural relevance as a nitrate transporter is poorly characterized. Yu et al. use the tools of *in silico* structural prediction of MtNPF1.7. They perform complementation tests by transforming the *Mtnpf1.7* mutant with a series of *MtNPF1.7* sequences harboring mutated amino acids that are predicted to be responsible for transport function. As a result, they identify the ExxE(R/K) motif in trans-membrane helices as being important for the nodulation-related role played by MtNPF1.7. They also identify the Motif A, which is conserved among major facilitator superfamily proteins, as essential for MtNPF1.7 transporter function and predict that this is important in other NPF transporters' functions. Given a structural similarity among NPFs, it is possible that the provided findings represent a useful insight into the future functional studies of other NPFs.

In conclusion, the information reported by the two reviews and the three original articles included in this Research Topic provide a very interesting picture of the nutrient signaling pathways governing different steps of the nodulation process. It is not by chance that most of the information provided here rely on the roles played by nutrient transporters (in this case mainly nitrate, phosphate and ammonium), as all the numerous transcriptomic analyses reported in literature, which refer to different steps of the symbiotic interaction, underline the central role played by transporters. This is particularly intriguing considering the potential action played by these proteins not merely as nutrient transporters, but either as nutrient sensors (transceptors) and their possible role in the crosstalk between external and endogenous signals (Lay-Pruitt and Takahashi, 2020).

## Author Contributions

All the authors have reviewed the contributions to the Research Topic, drafted the overall Editorial and approved it for publication.

## Conflict of Interest

The authors declare that the research was conducted in the absence of any commercial or financial relationships that could be construed as a potential conflict of interest.

## Publisher's Note

All claims expressed in this article are solely those of the authors and do not necessarily represent those of their affiliated organizations, or those of the publisher, the editors and the reviewers. Any product that may be evaluated in this article, or claim that may be made by its manufacturer, is not guaranteed or endorsed by the publisher.
